# Numerical computation and experimental assessment of a pressure-retaining gas-tight sediment sampler

**DOI:** 10.1038/s41598-024-65588-y

**Published:** 2024-06-24

**Authors:** Shudong He, Yangbin Xie, Haocen Bai, Youduo Peng, Yongping Jin

**Affiliations:** 1 School of Intelligent Manufacturing and Mechanical Engineering, Hunan Institute of Technology, Hengyang, 421002 China; 2 National-Local Joint Engineering Laboratory of Marine Resources Exploration Equipment and Safety Technology, Hunan University of Science and Technology, Xiangtan, 411201 China

**Keywords:** Gas-tight sampler, Sediment, Numerical computation, Pressure-retaining, Sea trial, Climate sciences, Ocean sciences

## Abstract

The pressure of the recovered sample is intricately connected to seawater temperature, the recovery velocity, and the pressure of the pre-charged gas. To better understand the sample pressure dynamics during the sampling recovery process, we focus on a gas-tight sediment sampler, delving into its pressure-compensation and pressure-retaining mechanisms. A comprehensive thermal and thermodynamic analysis is conducted throughout the entire pressure-retaining sampling process, examining the temporal variations in the temperatures of seawater and nitrogen within the sampler at various descending velocities. The heat transfer and thermodynamics are examined throughout the entire pressure-retaining sampling process to determine how changes in the temperatures of seawater and nitrogen, as well as the descent velocity, affect the pressure-retaining performance. The influence of pre-charging pressure and recovery velocities on the pressure-retaining performance of the sampler is examined. Then the proposed numerical model was well verified by the ultra-high-pressure vessel experiments of the sampler under 115 MPa. Finally, the sea trial results further verified the accuracy of the numerical model.

## Introduction

Scientists have found life at ocean depths greater than 6000 m and, due to the extreme environment, these organisms have attracted academic attention. Deep sediments contain large numbers of microorganisms and have become a treasure trove for natural products. Furthermore, studying deep-sea sediment samples is crucial for gaining an in-depth understanding of the sedimentary environments and geological history^1^. However, due to the high pressures of the deep-sea water layers, the exploration of abyssal organisms and their genetic resources remains insufficient, and our understanding of life in these extreme environments is limited^2–5^. Previous studies have shown that deep-sea sediments are rich in barophilic microorganisms. These microorganisms are adapted to the extremely high-pressure seabed environment and often exhibit barophilic growth characteristics necessitating high-pressure environments for survival^6^. When the pressure is reduced, for example, upon deep-sea samples are brought to the surface, consequences such as gas phase dissolution, component loss, organic decomposition, and the death of barophilic microorganisms can occur. Pressure-retained samples can preserve deep-sea conditions during collection, facilitating the study of deep-sea sediments. Indeed, pressure-retained samplers have facilitated the collection of abyssal microorganisms for modern medical research^7^. Therefore, improving the pressure-retaining rate of deep-sea sediment samplers has become a research focus in numerous institutions.

Pressure-retaining sediment samplers can collect sediments in their natural state, which is of broad scientific significance in the sampling and study of deep-sea sediments. At present, many types of samplers have been developed both domestically and internationally^8^, for example: the Deep Sea Drilling Program (DSDP) employs a pressure core barrel (PCB) ^9^ capable of operating at depths up to 3100 m, utilizing a ball valve at the lower end and closing an exhaust port at the cylinder’s apex. Deployed within the International Ocean Discovery Program (IODP), the pressure core sampler (PCS)^10^ employs a wire-line tool for hydraulic-driven sediment retrieval, achieving a maximum operational depth of 6900 m for superior pressure retention. The European Union’s hydrate autoclave coring equipment (HYACE)^11,12^, propelled by vibration, facilitates sample recovery from depths of up to 2500 m. The Japan Marine Geoscience and Technology Research Center has developed the hybrid pressure core sampler (hybrid PCS)^13^, which combines cable coring technology with an accumulator. It features a maximum operational depth of 3500 m, a core diameter of 51 mm, and can achieve a core length of up to 3.5 m. The German-engineered multiple autoclave corer (MAC)^14^ is capable of functioning at water depths ranging from 1000 to 2000 m, enabling the simultaneous deployment and operation of four pressure-retaining samplers within a single sampling operation. Developed in Japan, the pressure temperature core system (PTCS)^15^ is engineered to regulate core temperature and pressure, featuring a maximum operational pressure capacity of 24 MPa. It has been successfully deployed to a maximum water depth of 1006 m.

Pressure-retaining sediment samplers must perform two main functions, i.e., sampling and maintaining pressure. Sampling is the prerequisite for obtaining pressure-retained samples. Methods for collecting deep-sea samples have been extensively studied. For example, Skinner et al.^16^ established a mathematical model of the sampling process, which is based on the principles of soil mechanics and specifically addresses the influence of gravity samplers and piston samplers on sample disturbance. Henke et al.^17^ used a finite element analysis method to conduct the finite element simulations of an open pipe pile into the soil based on the large deformation theory. Zhou et al.^18^ established a two-dimensional axisymmetric finite element model of the contact between the sampling tube and the sediment, and studied the sediment disturbance during the sampling process with the circular tubular sampler. Qin et al.^19^ used an improved Drucker-Prager sediment constitutive model and proposed a numerical method for simulating gravity coring. The Euler-Lagrangian coupling method was used to analyze the sampling process, capturing under-sampling. Guo et al.^20^ adopted the discrete element model (DEM) to study the blocking mechanism of soil in pipe piles, and simulated the interactions between soil particles and between soil and pipe piles. Wegener et al.^21^ investigated the soil mechanical phenomena affecting the recovery of granular soils in tubular vibro flotation tests through literature review and correction of physical vibroflotation test results using the discrete element model. Chen et al.^22^ used ABAQUS software to analyze the sampling process of a proposed deep-sea sediment pressure-retaining device, then studied the influence of the installation position of the sampler and the diameter of the hole on the sediment sampling length. Ren et al.^23^ proposed a sampling depth optimization method based on structural design and parametric modeling for a self-floating sediment sampler, considering the influences of sampling depth, balance weight, ultimate stress friction coefficient, sampler size, and material properties on the sample length. Guo et al.^24^ used the Eulerian–Lagrangian method to study the sampling pipe-soil interaction for a self-developed pressure-retaining sampler. By studying the sampling process, the sampling pipe diameter for a satisfied sampling volume was obtained.

In addition to sampling technology, pressure-retaining technology is also a key technology for sediment sampling. Pressure-retaining technologies typically use compressed gas accumulators to compensate for pressure losses caused by volume changes due to leakage, deformation of the pressure vessel, and compression of sealing materials^25^. Pressure-retaining systems have been studied extensively, and a considerable number of publications on this topic exist. For example, Huang et al.^26^ studied the pressure-retaining performance of a sampler (working depth of 6000 m) using the accumulator and compensation chamber method, investigated the influence of gas volume on final pressure, and achieved good pressure-retaining performance. Huang et al.^27^ carried out theoretical analysis and modeling of pre-charged pressure changes in the sampler throughout the entire sampling process and determined the optimum value of the pre-charged gas, providing an important reference for setting the pre-charged pressure. To better understand the behavior of pre-charged gas under high pressure, Wang et al.^28^ derived a real gas equation of state based on the compressibility factor Z from experimental data and introduced a theoretical computation method to calculate the retained pressure and volume of the sample based on the gas equation of state^.^ Case et al.^29^ designed a new type of sediment sampler, which can be carried on an unmanned submersible for pressure-retaining sampling of sediment samples, with a maximum working pressure of 50 MPa. The sampler’s most distinctive feature is its ability to continuously supply liquid and gas to maintain constant pressure. Wang et al.^30^ carried out pressure experiments and sea trials on a new type of piston sampler designed with pressure compensation. The results showed that the sampler had good pressure-retaining capacity and could collect sediment samples from the deepest ocean in the world. Jackson et al.^31^ designed MAC-EXP and conducted deep-sea tests, and the results showed that the system reduced operating costs compared with other samplers in water depths of less than 3500 m.

Scientific research institutions and scholars tend to focus on the research and application of pressure-retaining samplers with water depths below 7000 m (and pressures less than 70 MPa). This inclination highlights significant gaps in the theoretical exploration of sample pressure-retaining technology, particularly regarding the mechanisms and procedures involved in ultra-high-pressure (up to 115 MPa) sampling. This paper examines the pressure-compensation and pressure-retaining mechanisms of a novel self-developed sediment sampler, performs heat transfer and thermodynamic calculations, and analyzes the entire pressure-retaining sampling process. Furthermore, this paper investigates how different pre-charged pressures and recovery velocities affect the retained pressures of samples, taking into account changes in seawater temperature. The correctness of the numerical model for pressure-retaining sampling has been confirmed through experiments. This work provides a theoretical foundation for the geometric design of the sediment sampler structure and the determination of operational parameters, laying the groundwork for addressing key issues related to efficient pressure maintenance.

## Principle of pressure-retaining sampling

### Numerical model

**Figure 1:**
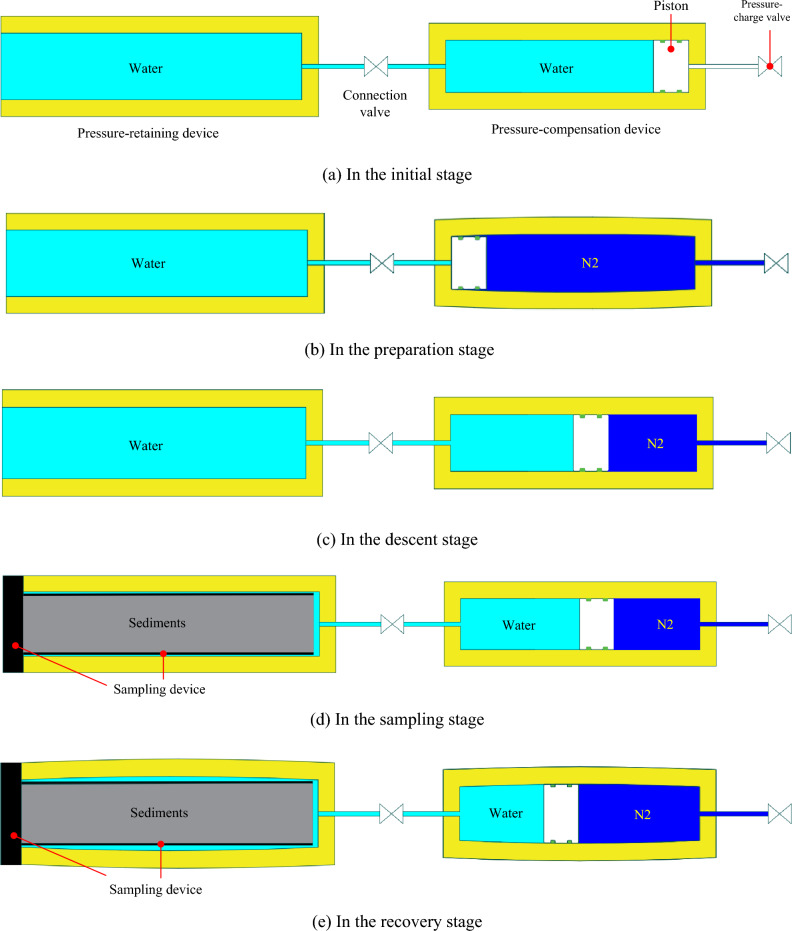
Schematic diagram of pressure compensation for the sampler.

The simplified schematic diagram of the pressure-compensation system is shown in Fig. 1. In the initial stage (Fig. 1a), the piston of the pressure-compensation device is at the rightmost end to ensure that the connecting valve is open. In the preparation stage (Fig. 1b), the pressure-compensation device is pre-charged to a certain pressure with nitrogen. In the descent stage (Fig. 1c), as the sampler descends with the submersible, the pressure in the pressure-retaining cylinder, which is connected to the outside seawater, increases continuously with increasing depth. Under the influence of the pressure differential, the pressure-compensation device continues to store energy. In the sampling stage (Fig. 1d), upon the submersible’s arrival at the sampling point, the manipulator grasps the sampling device and then inserts it into the pressure- retaining cylinder, ensuring the sealing of the pressure-retaining device. In the recovery stage (Fig. 1e), once the pressure-retaining cylinder and the sampling device are fully sealed, the pressure-compensation device uses highly-compressed nitrogen to release pressure energy, compensating for any pressure loss due to changes in ambient temperature and pressure differential.

### Nitrogen status during sampling process

Throughout the sampling process, from the sampler’s descent to the sampling point to its recovery after sampling, the pressure and temperature of the external seawater environment constantly change, affecting the pressure-retaining performance of the sampler. According to the changes in nitrogen pressure, volume, and temperature within the pressure-compensation device, the sampling process can be divided into four key stages: ① initial pressurization before deploying the sampler, ② descent of the sampler to the sampling point, ③ the act of sampling, and ④ recovery of the sampler to the ship’s deck.

The designed volume of nitrogen in the cylinder of the pressure-compensation device is denoted as *V*_C_. During process ①, upon completion of inflation, the pressure of the pre-charged nitrogen in the pressure-compensation device is *P*_0_, and the temperature of the nitrogen equals the ambient temperature, *T*_0_. The cavity of the pressure-compensation device expands due to the internal pressure, resulting in an internal volume slightly larger than the designed volume, denoted as *V*_C1_. During process ②, when one end of the cavity of the pressure-compensation device is opened to the external seawater, the pressure in the pressure-retaining cylinder of the pressure-retaining device increases with depth. The left and right pressure cavities of the piston achieve equilibrium when the pressure in the pressure-retaining cylinder equals the pre-charged nitrogen pressure. After this point, the piston is forced to the nitrogen side by the pressure differential, ensuring that the pressures inside and outside the charging cavity equal the increasing external seawater pressure. During this process, the nitrogen’s volume is compressed as the sampler descends to the sampling point.

During the descent, the nitrogen in the pressure-compensation device radiates heat to the outside seawater, and the temperature of the device drops. The nitrogen pressure at the sampling point is *P*_1_, the volume of nitrogen in the pressure-compensation device is *V*_1_, and the temperature of the nitrogen is *T*_1_. During process ③, the nitrogen pressure remains constant and equal to the external seawater pressure. While the submersible is at the sampling point, the nitrogen in the pressure-compensation device continues to exchange heat with the external seawater, causing the temperature to drop. When the nitrogen pressure in the pressure-compensation device is *P*_2_, the volume of nitrogen is *V*_2_, and the temperature of nitrogen is *T*_2_. During process ④, the pressure within the pressure-retaining device remains equal to the nitrogen pressure in the pressure-compensation device. As the external seawater pressure decreases, the pressure-retaining and pressure- compensation cylinders expand and deform due to the pressure differential, and the volume of nitrogen in the device increases to compensate for changes in sample pressure. Simultaneously, the nitrogen in the pressure-compensation device generates heat, which is then exchanged with the external seawater through the cylinder, resulting in a change in the nitrogen’s temperature. Upon reaching the sea surface, the nitrogen pressure in the pressure-compensation device is *P*_3_, the volume of nitrogen is *V*_3_, and the temperature of the nitrogen is *T*_3_.

This breakdown of the key processes reveals that there are three thermodynamic change processes occurring during stages ②, ③, and ④. During these three temperature change processes, the nitrogen within the pressure-compensation device contacts the cylinder’s inner wall and exchanges heat, which is subsequently exchanged with the external seawater. To determine the pressure and volume of nitrogen across these three key processes, it is essential to conduct heat transfer analyses for each of the distinct thermodynamic change periods and ascertain the corresponding nitrogen temperature values.

## Numerical computation model

### Seawater pressure

The pressure of seawater is not only directly related to the depth of the seawater, but also to its temperature, density, and salinity^32^. However, depth is the most critical factor affecting the pressure of seawater. Various methods exist to calculate seawater pressure, including the formula method, measurement method, empirical method, among others. For the convenience of calculation and analysis, the empirical formula proposed by McAllister and Myers^33^ is used here to calculate the pressure at the working depth of seawater:

1 $$P(h) = \left( {h + \frac{{0.3\left( {{\raise0.7ex\hbox{$h$} \!\mathord{\left/ {\vphantom {h {1000}}}\right.\kern-0pt} \!\lower0.7ex\hbox{${1000}$}}} \right)}}{0.44}} \right) \times {10}^{{ - 2}}$$

where: *h* is the depth of seawater, m; *P*(*h*) is the ambient pressure under the water depth(*h*), which takes into account the influence of the change in the elastic modulus on the density of seawater.

The velocity (*V*_sam_) of the submersible during descent and recovery in the vertical direction typically ranges from 20 to 60 m/min. Combined with the seawater pressure-depth curve, the expression for the ambient seawater pressure *P*(*τ*) as a function of time*τ*(min) is derived:

2 $$P(\tau ) = \left( {V_{{{\text{sam}}}} \cdot \tau + \frac{{0.3 \cdot \left( {{\raise0.7ex\hbox{${V_{{{\text{sam}}}} \cdot \tau }$} \!\mathord{\left/ {\vphantom {{V_{{{\text{sam}}}} \cdot \tau } {1000}}}\right.\kern-0pt} \!\lower0.7ex\hbox{${1000}$}}} \right)}}{0.44}} \right) \times {10}^{{ - 2}}$$

where *V*_sam_ is the submersible’s descent velocity, m/min; *P*(*τ*) is the seawater pressure at given moment *τ*, MPa; *τ* is the time, min.

### Distribution law of seawater temperature

**Figure 2:**
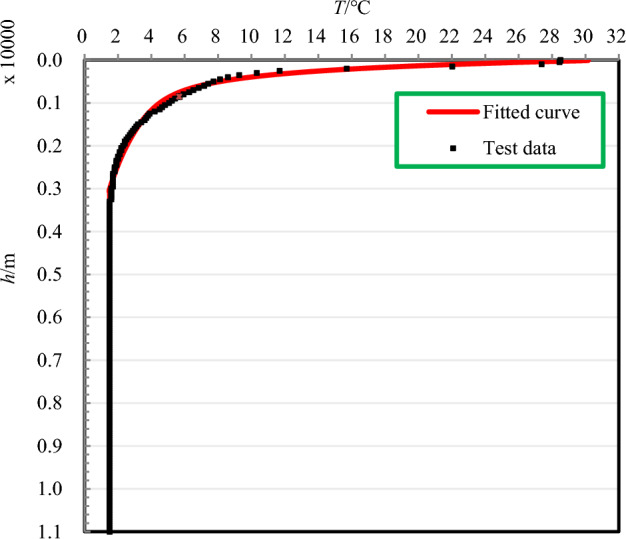
Temperature distribution curve of seawater.

The sampler is mounted on a submersible for both descent and recovery. During the descent, sampling, and recovery processes, the temperature of the external seawater at various depths dictates the temperature of the nitrogen within the pressure-compensation device. Thus, understanding the vertical temperature profile of seawater is essential for addressing the temperature changes within the nitrogen. The surface water temperature of the ocean typically ranges from − 2 to 30 °C, with an annual mean of about 17 °C. The Pacific Ocean is the warmest, with an average temperature of about 19 °C, whereas the Atlantic Ocean averages 16.9 °C. From the sea surface down to the main oceanic thermocline, the water temperature decreases markedly. The depth of the main thermocline varies by latitude, ranging from approximately 300 m to 800 m. An assumed depth of 400 m is used in this analysis. Below the main oceanic thermocline, the water temperature decreases gradually with increasing depth, albeit with a very weak gradient. Below 1,000 m, the water temperature changes minimally with depth, and below 3,000 m, it essentially remains constant at approximately 1.5 °C. Based on the measured temperature data from the research vessel “TANSUO-1” during its third expedition to the Mariana Trench in March 2017, with the sea surface temperature at approximately 30 °C, the vertical distribution of seawater temperature was approximated by a simplified function (3). The temperature distribution profile is depicted in Fig. 2.

3 $$T_{{\text{w}}} {\text{(h)}} = \left\{ {\begin{array}{*{20}l} {24.19exp( - 4.384 \times 10^{ - 3} h) + 7.02\exp ( - 5.122 \times 10^{ - 4} h)} \hfill & {h \in (0\;\;3300)} \hfill \\ {1.5} \hfill & {h \in [3300\;\;11000]} \hfill \\ \end{array} } \right.$$

where *h* is the seawater depth, m; *T*_w_(*h*) is the seawater temperature corresponding to that depth, °C.

When the water depth was less than 100 m, the seawater temperature remained above 30 °C, however, upon reaching 150 m, the temperature of the seawater began to drop rapidly. At a water depth of approximately 300 m, the seawater temperature had decreased to about 10 °C. The seawater temperature dropped to approximately 5 °C at a depth of about 1000 m. Below water depths of 3300 m, the seawater temperature was essentially constant at around 1.5 °C. Based on the distribution of seawater temperature, the variation of ambient seawater temperature *Tw*(h) over time τ(min) during the descent process can be expressed as:

4 $$T_{{\text{w}}} (\tau ) = \left\{ {\begin{array}{*{20}l} {24.19\exp ( - 4.384 \times 10^{ - 3} \cdot V_{{{\text{sam}}}} \cdot \tau ) + 7.02\exp ( - 5.122 \times 10^{ - 4} \cdot V_{{{\text{sam}}}} \cdot \tau )} \hfill & {\tau \in \left( {0\;\;\frac{55}{{V_{{{\text{sam}}}} }}} \right)} \hfill \\ {1.5} \hfill & {\tau \in \left[ {\frac{55}{{V_{{{\text{sam}}}} }}\;\;\frac{1}{{3V_{{{\text{sam}}}} }} \cdot 5500} \right]} \hfill \\ \end{array} } \right.$$

where *T*_w_(*τ*) is the seawater temperature at that moment*τ*, °C.

### Heat transfer and thermodynamic analysis

#### Mode of heat transfer

According to different heat transfer mechanisms, heat transfer primarily occurs through three pathways: heat conduction, heat convection, and heat radiation. One, two, or all three modes of heat transfer can simultaneously contribute to the heat exchange within a system, depending on the system’s complexity.

A. Heat conduction.

According to Fourier’s law, the heat flow rate per unit area per unit time (i.e., the heat flux) is directly proportional to the temperature gradient in the direction perpendicular to the heat transfer cross-section, and it is in the direction of decreasing temperature:

5 $$q = - \lambda_{k} \frac{{dT_{i} }}{dx}$$

where *q* is the heat flux density, W/m^2^;*λ*_*k*_ is the thermal conductivity coefficient, W/ (m⋅K);*T* is the temperature field along the* x-*axis direction;$\frac{{dT_{i} }}{dx}$ is the temperature gradient, K/m.

The seawater temperature varies with depth. This variation will affect the heat exchange between the nitrogen in the pressure-compensation device and the seawater throughout the sampling process, from the descent to the sampling point until recovery at the sea surface. The temperature distribution of nitrogen can be characterized as a function of time and spatial coordinates. For the three-dimensional, unsteady-state heat transfer problem without an internal heat source, the heat conduction differential equation is derived using the law of conservation of energy and Fourier’s law:

6 $$\frac{\partial T}{{\partial \tau }} = \frac{{\lambda_{k} }}{{\rho {\text{c}}}}\left( {\frac{{\partial^{{2}} T}}{{\partial x^{2} }} + \frac{{\partial^{{2}} T}}{{\partial y^{2} }} + \frac{{\partial^{{2}} T}}{{\partial z^{2} }}} \right)$$

The expression in the cylindrical coordinate system is as follows:

7 $$\frac{\partial T}{{\partial \tau }} = \alpha \left( {\frac{{\partial^{{2}} T}}{{\partial r^{2} }} + \frac{1}{r} \cdot \frac{{\partial^{{2}} T}}{\partial r} + \frac{1}{{r^{2} }} \cdot \frac{{\partial^{{2}} T}}{{\partial \varphi^{2} }} + \frac{{\partial^{{2}} T}}{{\partial z^{2} }}} \right)$$

8 $$\alpha = \frac{{\lambda_{k} }}{{\rho {\kern 1pt} {\kern 1pt} c}}$$

where *λ*_*k*_* is* the thermal conductivity coefficient, W/m⋅K;*ρ* is the material density of the sampler, kg/m^3^;*c* is the specific heat capacity of the sampler material, kJ/(kg⋅℃);*T* is the temperature of a certain point in space, °C;*α is* thermal diffusivity coefficient, m^2^/s;*τ* is the time coordinate, s; $r,\varphi ,z$ are the cylindrical coordinate components.

B. Thermal convection.

The sampler is mounted within the sampling basket of the submersible. Throughout the sampling process, convective heat transfer occurs between the seawater and the cylinder wall of the pressure-compensation device; Thus, the Newtonian cooling convection heat transfer equation is applicable:

9 $${\text{Q}} = {\text{h}}_{{\text{f}}} \cdot {\text{S}} \cdot \left| {T_{{\text{S}}} - T_{{\text{B}}} } \right|$$

where h_f_ is the surface convective heat transfer coefficient, W/m^2^⋅K; S is the surface area available for convective heat transfer, m^2^; *T*_S_ is the solid surface temperature, K; *T*_B_ is the bulk fluid temperature, K.

Given that the influence of seabed thermal radiation on the sampler is negligible, the effect of radiation on the sampler can be disregarded. This implies that only conductive and convective heat exchanges must be considered in the calculations.

### Boundary conditions

The heat transfer coefficient and the temperature of the seawater flowing over the sampler cylinder’s surface are known and can be expressed as follows:

10 $$- \lambda_{{\text{k}}} \frac{{{\text{d}}T}}{{{\text{dr}}}} = {\text{h}}_{{\text{f}}} \cdot \left| {T - T_{B} } \right|$$

where *T*_B_ is the temperature of the bulk fluid, K; h_f_ is the heat transfer coefficient, W/m^2^⋅K.

The temperature of the nitrogen within the sampler stabilizes at approximately 1.5 °C upon reaching the seabed and is nearly equal to the sea surface temperature of about 30 °C upon recovery to the sea surface.

**Figure 3:**
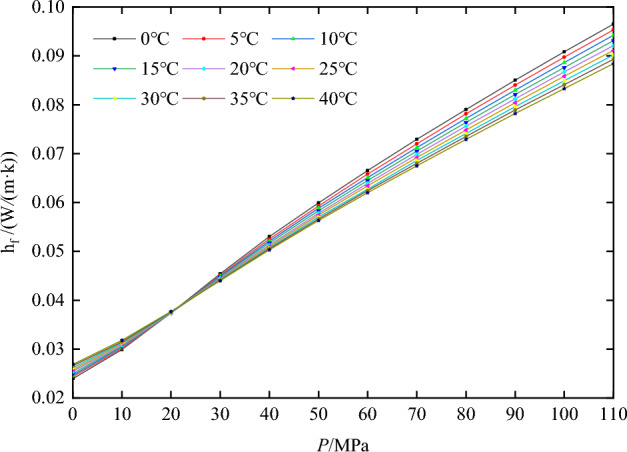
Thermal conductivity of nitrogen at various temperatures and pressures.

**Figure 4:**
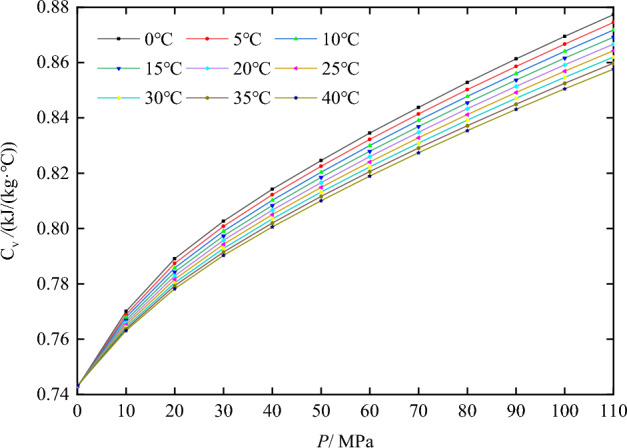
Constant-volume specific heat capacity of nitrogen at various temperatures and pressures.

The transient temperature of the pressure-compensation device is analyzed during the descent, sampling, and recovery stages using the finite element method. First, the physical parameters of the cylinder material and nitrogen are established. The physical parameters of the TC4 titanium alloy are as follows: density(*ρ*)is 4.51 × 10^3^ kg/m^3^, Poisson’s ratio (*μ*)is 0.34, elastic modulus (*E*) is 1.13 × 10^5^ MPa. The wall convective heat transfer coefficient(*α*) is 100W/m^2^⋅K, and the thermal conductivity(*λ*_*k*_) is 7.955 W/m⋅K. Figures 3 and 4 show the thermal conductivity coefficient (h_f_) and constant-volume specific heat capacity(C_v_)of nitrogen under various temperatures and pressures.

## Results and discussion

### Temperature changing over seawater depth under different descent velocities

**Figure 5:**
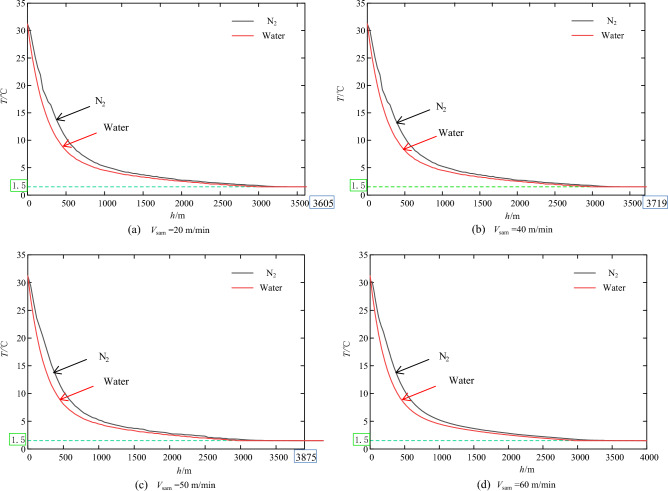
Temperature variation with seawater depth under different descent velocities.

As the sampler descends, the pressure within the pressure-compensation device increases until equilibrium is reached on both sides of the piston; whereupon the piston is forced to the nitrogen side by the pressure differential, compressing the nitrogen gas. Concurrently, the nitrogen dissipates heat to the surrounding seawater via the cylinder wall, resulting in a temperature decrease. For analytical and computational convenience, an initial temperature (*T*_0_) of 31 °C was assumed at the sea surface. The temperature change curves of the sampler with respect to seawater depth were calculated for various descent velocities, as depicted in Fig. 5. As shown in Fig. 5, the decrease in nitrogen temperature with increasing depth closely mirrors the external seawater temperature profile across all velocities. It exhibits a sharp decline from 0 to 1000 m, then plateaus between 1000 to 4000 m, and stabilizes at approximately 1.5 °C at depths beyond 4000 m. At equivalent seawater depths, the nitrogen temperature is marginally higher than the seawater temperature, indicating continuous heat exchange between the nitrogen and the surrounding seawater. Furthermore, at descent velocities of 20 m/min, 40 m/min, 50 m/min, and 60 m/min, the nitrogen temperature equated with the external seawater at depths of 3605 m (Fig. 5a), 3719 m (Fig. 5b), 3875 m (Fig. 5c), and 4000 m (Fig. 5d), respectively. Throughout the descent, nitrogen and seawater continuously exchange heat, with both temperatures ultimately converging to approximately 1.5 °C; therefore, the temperature change profiles from 4000 m to 11,000 m are excluded from the figure. These calculations demonstrate that, with a known descent velocity, the depth at which the nitrogen and seawater temperatures essentially equate can be determined using the heat transfer model.

Due to changes in pressure and seawater temperature, the nitrogen volume and temperature decrease as the sampler descends, thereby increasing the nitrogen pressure to match the pressure at the sampling point. Using heat transfer analysis, the nitrogen volume (*V*_1_) at the sampling point when the sampler arrives can be determined. This volume is related to the sampler’s structural dimensions, the pre-charged pressure, and the external pressure and temperature at the sampling point.

During sediment sampling, the pressure at the sampling point can be calculated based on the distribution of external seawater temperatures at various depths. Upon completion of the sampling operation, if the nitrogen temperature exceeds the ambient temperature at the sampling point, the volume of nitrogen (*V*_2_) must be calculated. If the nitrogen temperature equals the ambient temperature at the sampling point, then *T*_1_ = *T*_2_ and *V*_1_ = *V*_2_. Consequently, the nitrogen temperature at the conclusion of sampling is dictated by the sampler’s descent velocity and the sampling depth.

**Figure 6:**
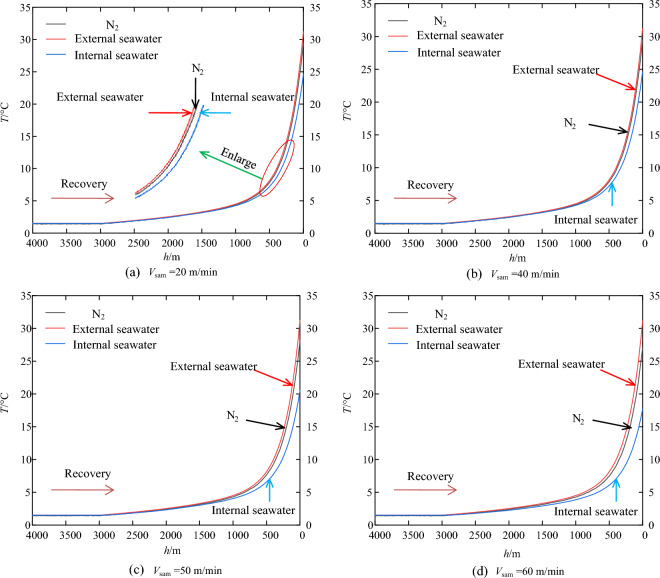
Temperature variation with seawater depth under different recovery velocities.

During the recovery stage, heat transfer between the external seawater and the nitrogen within the pressure-compensation device is contingent upon the temperature differential between the two. Given the maximum operating depth of 11,000 m at the sampling point, the temperature profiles of seawater and nitrogen within the sampler during the recovery phase, at recovery velocities of 20 m/min, 40 m/min, 50 m/min, and 60 m/min, have been calculated and are depicted in Fig. 6 (temperatures remain unchanged from depths of 11,000 m to 4000 m and are thus excluded from the figure).

The figure shows that, within the sampler, nitrogen and seawater temperatures follow a trajectory that closely resembles the external seawater temperature profile, with a gradual rise from 11,000 m to 1000 m, followed by an abrupt ascent to sea level. At equivalent depths, the nitrogen consistently registers a higher temperature than the enclosed seawater, yet both are invariably below the external temperature. This suggests continuous heat transfer with the external environment. Given nitrogen’s thermal diffusivity is significantly greater than that of water, it is anticipated that nitrogen will equilibrate to temperature changes more swiftly within the sampler.

**Figure 7:**
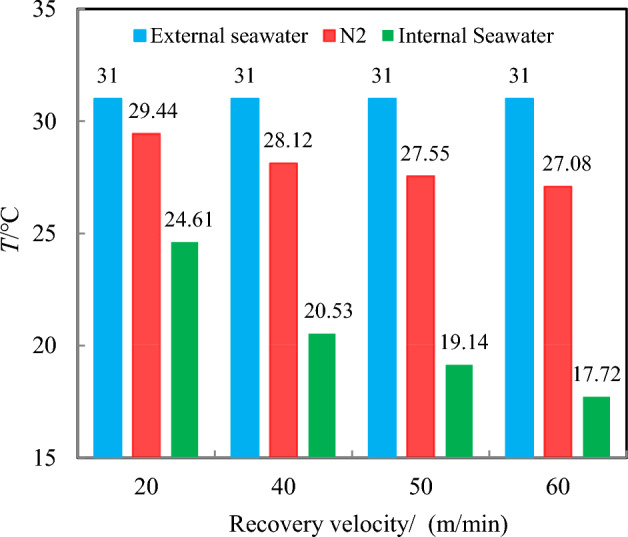
Temperature distribution as the sampler is recovered to the deck.

Furthermore, due to differences in diffusivity, as the sampler ascends to the sea surface, the temperature difference between nitrogen and external seawater differs from that between internal seawater and external seawater, and the temperature difference increases with the increase of recovery velocity. However, the recovery velocity has a relatively minor effect on the nitrogen temperature variation, as depicted in Fig. 7. For instance, at an recovery velocity of 20 m/min, the average temperatures of the internal seawater and nitrogen are 24.61 °C and 29.44 °C, respectively. Upon increasing the recovery velocity to 60 m/min, the average temperatures of the internal seawater and nitrogen are 17.72 °C and 27.08 °C, respectively, representing a decrease of 6.89 °C and 2.36 °C, respectively. Additionally, because of nitrogen’s high thermal diffusivity, its final temperature closely approximates that of the external seawater temperature, and the smaller the velocity, the more pronounced this trend becomes. For example, at a recovery velocity of 20 m/min, the temperature difference between the two is merely 1.56 °C.

### Influence of pre-charged pressure on retained pressure

**Figure 8:**
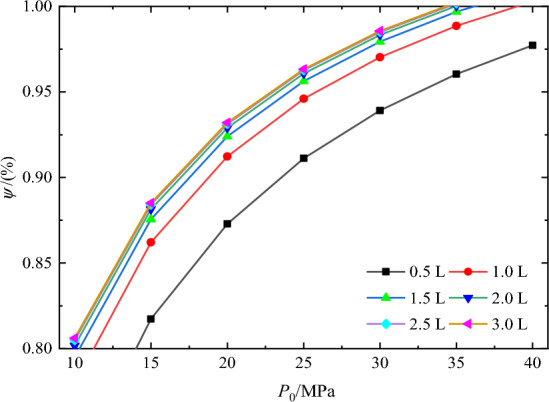
Influence of pre-charging pressures on pressure-retaining pressure.

Taking an operating water depth of 11,000 m, which corresponds to a maximum working pressure of 115 MPa, as an example, the retained pressure and pressure-retaining rates of the sample post-sampling are calculated. Figure 8 shows the influence of pre-charge pressure on the pressure-retaining rate of the sampler under temperature differences of 0 °C and 25 °C, respectively. It is evident from the figure that the pressure-retaining rate increases with increasing pre-charge pressure when the working volume remains constant, exhibiting an approximately linear trend.

For instance, when the working volume is 0.5 L, the pressure-retaining rate rises from 80.2 to 96.8% as the nitrogen pre-charge pressure increases from 10 to 40 MPa, marking an increase of 20.7%. Consequently, increasing the pre-charge pressure significantly affects the pressure-retaining performance of the sample. Figure 8 indicates that at working volumes of 1 L, 1.5 L, 2.0 L, 2.5 L, and 3.0 L, and under a temperature difference of 25 °C, the maximum pressure-retaining rate can attain 100% with increased pre-charge pressure. This suggests that as nitrogen is heated and expands more significantly with greater temperature differentials, the internal pressure within the sampler exceeds the pressure at the sampling point. It is apparent that the pre-charge pressure significantly influences the sample’s pressure-retaining rate. Given nitrogen’s high compressibility and the associated risks, the sampler’s design must ensure that the materials and construction are robust enough to satisfy safety requirements. Moreover, the pre-charge pressure ought to be as high as possible to maximize the sampler’s pressure-retaining performance. This approach is viable, as increasing the pre-charge pressure is both straightforward and effective in practical operations.

### Influence of recovery velocity on retained pressure

The recovery velocity of the sampler affects the temperature of the seawater and nitrogen within it. Using the heat transfer mathematical model, the pressure of nitrogen at various times can be calculated. This calculated pressure represents the retained pressure of the sample. It is clear that the recovery velocity of the sampler impacts its pressure-retaining performance. Considering this fact, this section uses the maximum operating depth of 11,000 m as an example to examine the effects of temperature differences and to determine the pressure loss within the sampler across various recovery velocities.

**Figure 9:**
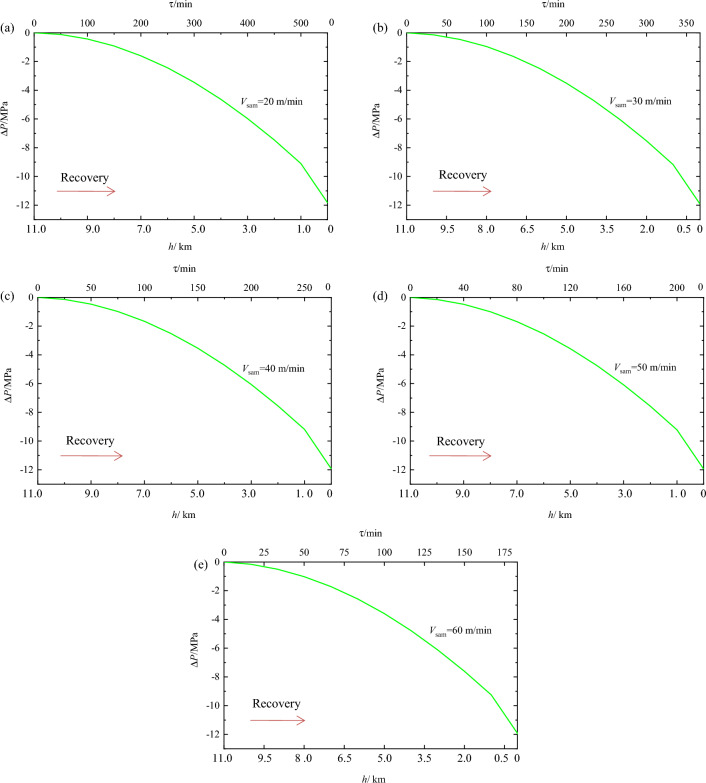
Influence of different recovery velocities on pressure-retaining pressure.

**Figure 10:**
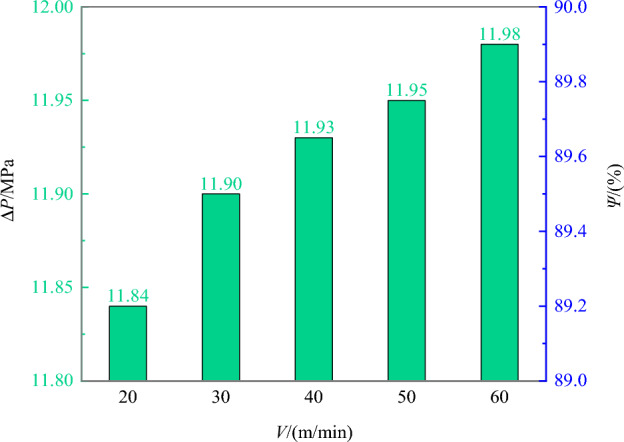
Final pressures and pressure-retaining rates of samples at different recovery velocities.

Figure 9 shows the sample pressure loss over time at recovery velocities of 20, 30, 40, 50, and 60 m/min. It is evident from the figure that the sample pressure loss in the sampler over time follows an approximately parabolic curve. Upon reaching the surface, the sample pressure loss ranged from 11.84 MPa to 11.98 MPa across the various recovery velocities. As shown in Fig. 10, it is observed that the pressure loss increases with increasing recovery velocity. For instance, when the recovery velocity rose from 20 m/min to 60 m/min, marking a 200% increase, the pressure loss grew from 11.84 MPa to 11.98 MPa, resulting in a 0.14 MPa increase in pressure loss and a 0.12% decrease in the pressure-retaining rate. Nonetheless, although reducing the recovery velocity can decrease the sample pressure loss and thus enhance the pressure-retaining rate, the recovery velocity’s impact on the final pressure and pressure-retaining rate of the sample is relatively minor. Thus, the sampling recovery velocity can be adjusted based on other operational requirements.

## Pressure-retaining experiment

### Principle of experiment

**Figure 11:**
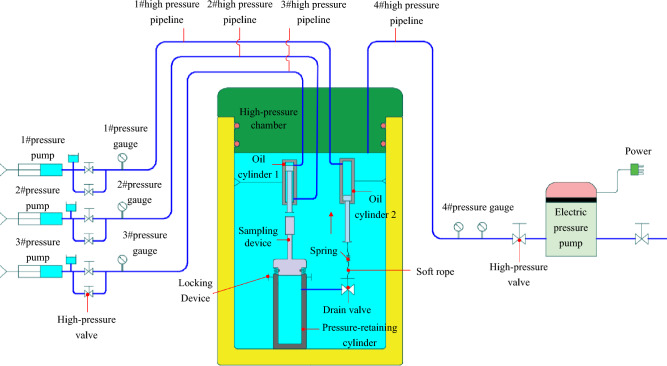
Schematic diagram of pressure-retaining test.

In this experiment, the sediment sampler was selected as the research subject, and the overall plan for the experimental system was established. The basic working principle is depicted in the overall schematic of the experimental system in Fig. 11: Initially, the sampler and oil cylinders are positioned within the high-pressure chamber, connected to the appropriate pipelines, and the chamber is pressurized to the desired experimental pressure using an electric pressure pump. Subsequently, the sampler is sealed at ultra-high pressure by actuating the pressure pump, which performs the sampling and valve closure actions of the sampling device. Following that, the high-pressure valve on the electric pressure pump is opened to gradually depressurize the high-pressure chamber. Ultimately, the sampler’s pressure-retaining performance is confirmed by measuring the pressure within the pressure-retaining cylinder.

### .The experimental system

The experimental system primarily consists of three components: a high-pressure chamber system, oil cylinder action systems, and the sampler. The high-pressure chamber system comprises a high-pressure chamber cylinder, an electric pressure pump, high-pressure valves, pressure gauges, and high-pressure pipelines. The primary function of the system is to provide a pressure-resistant, enclosed space and the necessary pressure environment for the experiment. The maximum experimental pressure can reach 115 MPa. The oil cylinder action systems mainly include sampling action oil cylinder 1, oil cylinder 2, valve-closing action oil cylinders, and their associated control pipelines, among other components.

**Figure 12:**
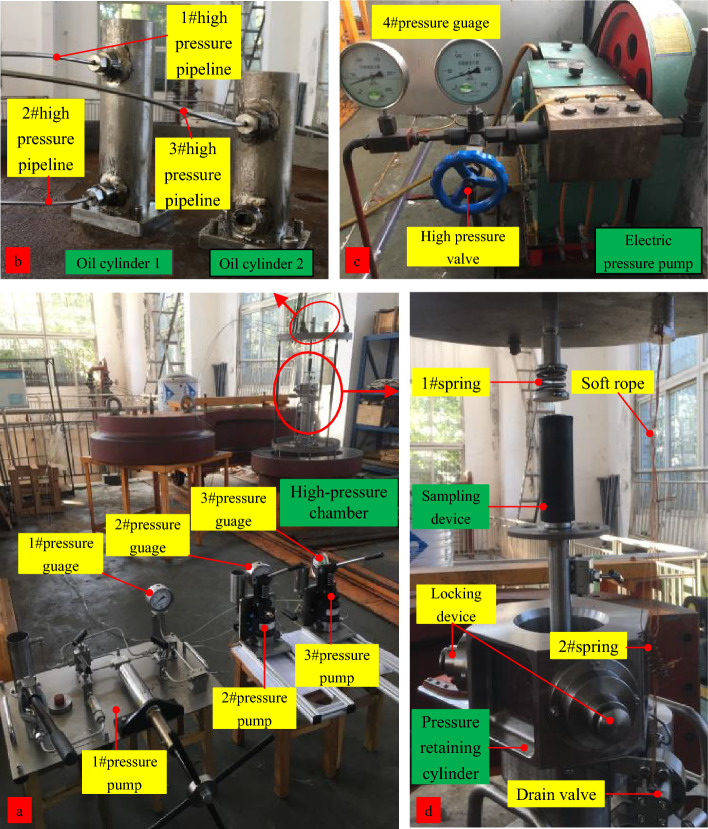
Pressure-retaining test.

The sampling action oil cylinder 1 and the valve-closing action oil cylinder 2 are secured to the specialized bench. A number 1 spring is installed at the lower end of the piston rod of the sampling action oil cylinder 1 to provide cushioning as the piston rod moves downward. The number 1 high-pressure pipeline is connected to the number 1 pressure pump and the number 1 high-pressure port, the number 2 pipeline to the number 2 pump and port, and the number 3 pipeline to the number 3 pump and port. The lower end of the piston rod of the valve-closing action oil cylinder 2 is attached to the sampler’s drain valve using a flexible cord. A number 2 spring is positioned midway along the flexible cord to provide cushioning upon upward piston rod movement. The experimental system is illustrated in Fig. 12:

### Experimental procedures

**Figure 13:**
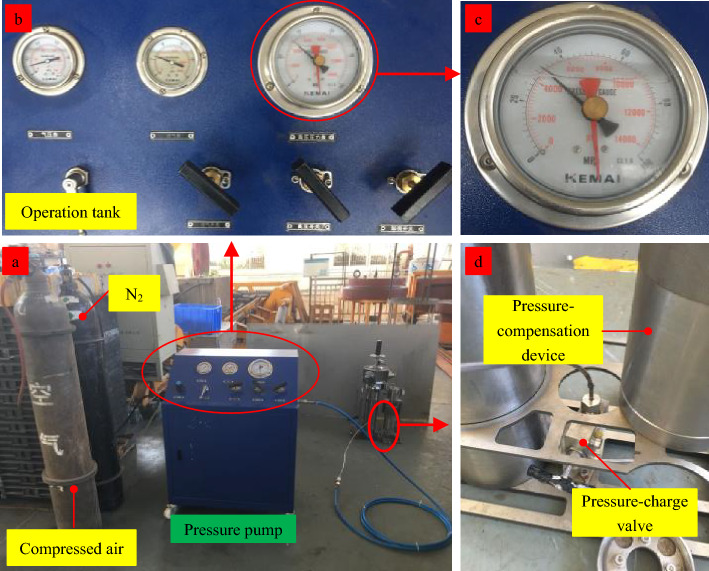
Pre-charging of pressure-compensation device.

**Figure 14:**
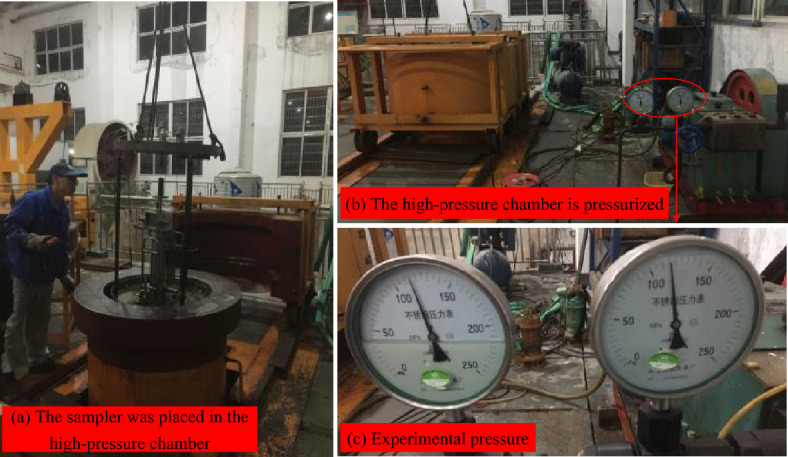
Pressure-retaining in high pressure chamber.

During the experiment, the sampler is secured to the specialized bench and then placed into the high-pressure chamber for the pressurization test. The pressure within the chamber is raised to the test pressure of 115 MPa, and subsequently, a hydraulic cylinder is utilized to propel the sampling tube, simulating the sampling action until the sampler is both locked and sealed. Once the pressure has been maintained for the designated duration, it is then released, the sampler is extracted and inspected for any signs of damage or deformation, followed by measuring and recording the sample pressure within the pressure-retaining cylinder. The specific steps are as follows:

1. As shown in Fig. 13, the low-pressure inlet of the pressure pump is connected to the compressed air tank and the nitrogen cylinder, and the high-pressure outlet is linked to the charging valve. Utilizing the pressure pump, nitrogen is pre-charged into the pressure-compensation device to a specific pressure via the pre-charging valve, following which the pre-charging valve is secured.
2. Trial operation. The sampler is secured to the specialized bench and positioned appropriately above the pressure-retaining cylinder. First, the number 1 pressure pump is actuated to position the piston of the sampling cylinder at the top. Subsequently, the pressure pump is operated to place the piston of the valve-closing oil cylinder 2 at the lower end and to open the drain valve via the number 4 pressurizing port. Then, the number 1 pressure pump is actuated to drive the piston rod of the sampling cylinder downward, applying pressure to the sampling device until locking and sealing are achieved. Following this, the number 3 pressure pump is actuated to manipulate the piston rod, initiating the upward cylinder’s closing action until the drain valve is sealed.
3. After verifying that the pipelines are connected, the gantry crane is employed to gradually raise the sampler, along with the specialized bench, into the high-pressure chamber. The upper cover is then sealed, and pressurization preparations are undertaken after confirming the absence of leaks in the seals (Fig. 14a).
4. The number 3 pressure pump is actuated to pressurize the chamber until the gauge reading of the number 3 gauge slightly exceeds the experimental pressure. The power supply for the high-pressure chamber’s pressurization system is then engaged to gradually achieve the experimental pressure, sustained for 5 min (Fig. 14b and c).
5. As pressure increases, the pointer of the number 1 pressure gauge will swing to the left. As the gauge continues to register increasing pressure, the piston extends further toward the cylinder’s lower end to maintain constant pressure.
6. The number 1 pressure pump is operated to depressurize, while the number 2 pressure pump is operated to pressurize. Concurrently, the piston rod of the sampling action oil cylinder ascends to the top position, and the locking mechanism secures the sampling device.
7. The number 3 pressure pump is operated to gradually release the pressure. At this juncture, the piston rod of the valve-closing action cylinder, connected to the drain valve, rises due to the differential hydrostatic pressure within the high-pressure chamber, thereby closing the drain valve and sealing the sampler.
8. The pressure-release valve of the high-pressure chamber system is actuated to vent the pressure. Once the pressure has been relieved, the high-pressure chamber is opened, and the experimental bench is extracted to the specified position. The pressure within the pressure-retaining cylinder is then measured.

### The experimental results

**Figure 15:**
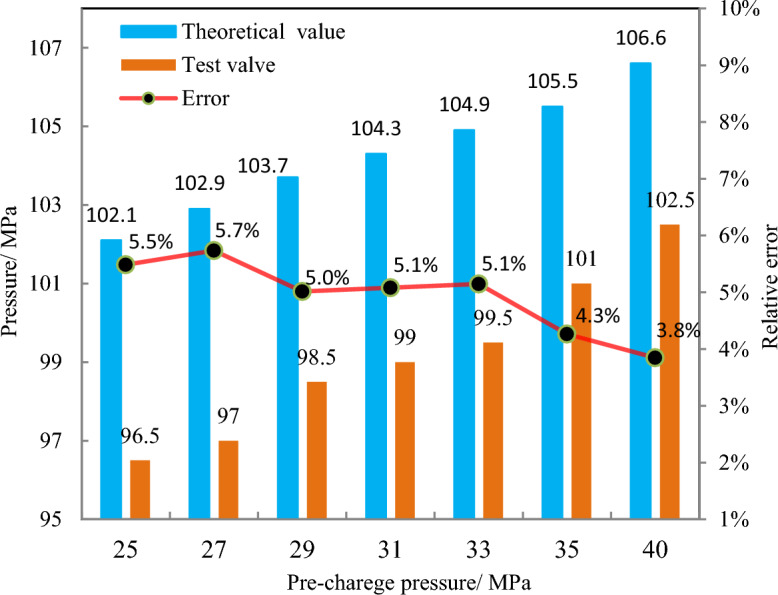
Analysis of the experimental results.

The pressure-compensation device was pre-charged with nitrogen at pressures of 25 MPa, 27 MPa, 29 MPa, 31 MPa, 33 MPa, 35 MPa, and 40 MPa, and experiments were conducted. To account for experimental error, three repetitions were performed at each pressure level, and the mean value was adopted as the experimental result. Throughout the experiment, it was ensured that the sampler was free of leakage, damage, and deformation. The outcomes of the experiments are presented in Table 1. Figure 15 illustrates that the final pressures of the sampler increased with increasing pre-charge pressure under the same conditions, but they were lower than the theoretical values. The upward trend of the experimental values was generally consistent with the theoretical values, with an average relative error of 4.9%. The experimental findings further confirmed the accuracy of the model.

**Table 1:** Results of the pressure-retaining test.

Pre-charged pressure /MPa	25	27	29	31	33	35	40	Average
Theoretical value /MPa	102.1	102.9	103.7	104.3	104.9	105.5	106.6	104.3
Test value /MPa	96.5	97.0	98.5	99.0	99.5	101.0	102.5	97.6
Relative error /(%)	5.5	5.7	5.0	5.1	5.1	4.3	3.8	4.9

## Sea trials

**Figure 16:**
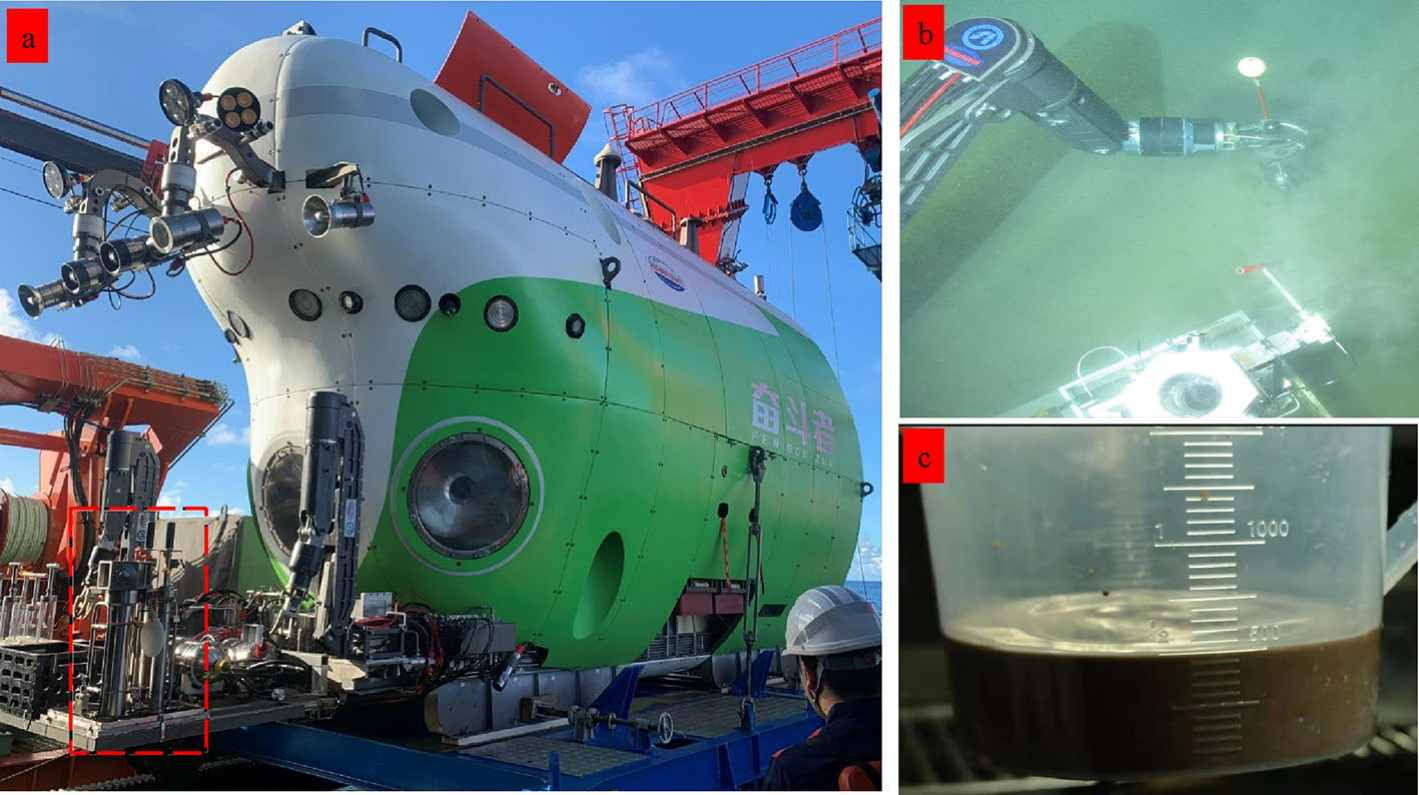
Sites of sea trials.

The sampler underwent field testing in the abyss of the West Philippine Basin during the TS-21 expedition, organized by the Institute of Deep-Sea Science and Engineering, Chinese Academy of Sciences. The sampler was deployed thrice on the FENDOUZHE manned submersible from the research vessel “Tansuo-1”. Pressure-retaining sediment samples were successfully retrieved from the 7700 m deep seafloor. The deployments of the sampler and the sample collections are depicted in Fig. 16.

During the initial sea trial, due to the drain valve’s failure, the pressure-retaining sampling action was unsuccessful. Following improvements to the sampler’s drain valve in the field, it was confirmed that pressure-retained sediment samples were successfully collected during the second and third operations. The final pressures recorded were 74.5 MPa and 81.0 MPa, respectively. During the third sampling process, the pressure of the final sample exceeded the in-situ pressure because the submersible’s temperature upon surfacing being higher than its initial temperature at depth, leading to gas expansion. These findings indicate that the sampler is effective at acquiring high-quality pressure-retained sediment samples from abyssal environments at depths exceeding 7,000 m.

## Conclusion

1. During the descent, the nitrogen temperature mirrors external seawater temperature, dropping sharply to 1000 m. Below this depth, it stabilizes at 1.5 °C. Notably, the nitrogen temperature remains slightly higher than the surrounding seawater from 0 to 4000 m.
2. During recovery, nitrogen and seawater temperatures in the sampler track the external seawater temperature profile, rising gradually from 11,000 m to 1000 m, then increasing sharply below 1000 m. Nitrogen consistently records higher temperatures than the enclosed seawater but is cooler than the external environment. The temperature difference between nitrogen and external seawater changes with recovery velocity, minimally affecting nitrogen temperature variation. For example, at 20 m/min, this difference is only 1.56 °C.
3. Increasing the pre-charge pressure can significantly enhance the sampler’s pressure-retaining rate. Provided that safety requirements are met, the pre-charge pressure should be appropriately increased. It is recommended to maintain an optimal pre-charge pressure of 20 to 40 MPa to substantially improve the pressure-retaining rate.
4. The experimental results were in close agreement with the theoretical values, with an average discrepancy of 4.9%. The sea trial data further showed that the sampler can acquire high-quality, pressure-retained sediment samples from abyssal environments at depths exceeding 7000 m.

## Data Availability

The datasets used and analysed during the current study available from the corresponding author on reasonable request.
